# Expression of Concern: “Plaque Begets Plaque. ApoB Does Not: Longitudinal Data from The KETO-CTA Trial” [JACC: Advances, Volume 4, Issue 7, July 2025, Article 101686]

**DOI:** 10.1016/j.jacadv.2026.102607

**Published:** 2026-01-23

The Editors of *JACC: Advances* wish to inform readers that concerns have been raised regarding the integrity of data and/or analyses presented in the paper mentioned above. These concerns are currently under confidential review in accordance with the *Journal*’s editorial policies and the Committee on Publication Ethics (COPE) guidelines. While this process is ongoing, the Editors believe it is important to alert readers to the existence of these concerns

